# Roles of NET Peptides With Known Antimicrobial Activity and Toxicity in Immune Response

**DOI:** 10.1155/jimr/5528446

**Published:** 2024-12-27

**Authors:** Sinan Cebeci, Tuba Polat, Nihan Ünübol

**Affiliations:** ^1^Department of Medical Biotechnology, Institute of Health Sciences, Acibadem Mehmet Ali Aydinlar University, Istanbul, Türkiye; ^2^Department of Medical Microbiology, School of Medicine, Acibadem Mehmet Ali Aydinlar University, Istanbul, Türkiye; ^3^Medical Laboratory Technician Program, Vocational School of Health Services, Acibadem Mehmet Ali Aydinlar University, Istanbul, Türkiye

**Keywords:** antimicrobial peptides, IFN-*γ*, IL-6, macrophage, TNF-*α*

## Abstract

Antimicrobial peptides (AMPs) are crucial components of the innate immune system in all living organisms, playing a vital role in the body's defense against diseases and infections. The immune system's primary functions include preventing disease-causing agents from entering the body and eliminating them without causing harm. These peptides exhibit broad-spectrum activity against bacteria, viruses, fungi, parasites, and cancer cells. They are secreted by innate and epithelial cells and contribute to host defense by inducing cellular activities such as cell migration, proliferation, differentiation, cytokine production, angiogenesis, and wound healing. In response to the growing challenge of bacterial resistance to antimicrobial agents, alternative drugs and new antibacterial molecules are being explored. In a previous study, NET AMPs were synthesized and their antimicrobial effects were determined. The current study extends this work by assessing the effects of these peptides on the immune system through cell culture experiments and ELISA. Specifically, the study investigated how different concentrations of these peptides influence the secretion of interleukin-6 (IL-6), tumor necrosis factor-*α* (TNF-*α*), and interferon-*γ* (IFN-*γ*) in mouse macrophages. Among the synthesized peptides, NET1 and NET2 demonstrated low cytotoxicity in TIB-71 RAW 264.7 macrophages. These peptides induced an anti-inflammatory response and reduced IL-6 expression in the absence of LPS stimulation, while simultaneously increasing IFN-*γ* and TNF-*α* secretion. These findings suggest that NET1 and NET2 peptides possess both anti-inflammatory and pro-inflammatory properties, highlighting their potential role in modulating immune responses.

## 1. Introduction

In recent years, the increase in irregular use of antibiotics around the world has become unstoppable. Antibiotics have become commonplace, routinely used in medical practice. Consequently, resistance mechanisms in microorganisms have developed, enhancing their ability to survive by disrupting the action pathways of antibiotics. As a result, antibiotics used to treat many diseases have become insufficient, leading to a search for alternative treatments. Many scientists are now exploring innovative treatment areas and new molecules. Our environment harbors numerous microorganisms, many of which are pathogenic and highly dangerous to human health. These pathogens have developed significant resistance mechanisms against antibiotics, and at this point, antimicrobial peptides (AMPs), potent and naturally and synthetically occurring structures, are emerging as a defense against these detrimental consequences [1, 2].

AMPs are naturally and synthetically occurring structures that are produced and synthesized by many living organisms, including humans, which act against microbial sources. These structures play a crucial role in the innate immune system, contributing to nonadaptive defense mechanisms against various unsanitary microorganisms. In addition, another advantage of AMPs is that they exhibit broad-spectrum antimicrobial activity. Thus, they could be innovative therapeutic molecules with high effectiveness against many microorganisms [3–5].

Examples of AMPs found in nature include cathelicidins and defensins. These peptides are produced by mammals. Cathelicidins are positively charged peptides [6]. Their mechanism of action involves direct effects on the cell membrane. Specifically, they interact electrostatically with the cell membrane and create pores, leading to death of the microbe [3, 7]. LL-37, AMP, and cathelicidin derivative is produced in humans. This peptide, which has an *α*-helical structure, penetrates the cell membrane and directly kills the microorganism by disrupting the cell membrane [8, 9].

In previous studies by Polat et al. [10], LL-37-like peptides were designed, their amino acid sequences were synthesized, and their antimicrobial activity and cytotoxicity results were published. These NET peptides were rendered resistant to protease and their activity was tested on different biofilm forming bacteria [11]. Determination of the effect of these designed peptides on macrophages are revealed for the first time in our study. Understanding the role of peptides in the immune system is crucial for the future development of these peptides as new drug molecules.

## 2. Materials and Methods

### 2.1. Design of AMPs, Solid-Phase Chemical Synthesis, and High-Performance Liquid Chromatography (HPLC) Analyses

In the literature, AMPs are generally described as hydrophobic and positively charged [12]. In our previous study, we designed four hydrophobic and positively charged peptides of 10–20 amino acids in length using d- and l-form peptides with a similar structure as LL-37, which we found to be effective against bacterial biofilms [10]. In this study, we examined the effects of these four peptides on macrophages to clarify their effects on the immune system (Table 1).

The peptides were synthesized using a Liberty Blue peptide synthesizer (CEM, Matthews, NC, USA). After the synthesis was complete, the peptides were cleaved from the solid support using a Razor device, as specified in the manufacturer's manual. The synthesized AMP samples were first prepared at a concentration of 2 mg/mL for analysis by reversed-phase HPLC. For HPLC analysis, a C-18 analytical column was used, with Buffer A (dH_2_O + 0.05% TFA) and Buffer B (acetonitrile + 0.025% TFA) as the mobile phases. After equilibrating the column at 5% Buffer B, the peptide was injected and eluted using a gradient protocol (%20–%80 Buffer B). The peptides were run on an analytical HPLC column, and their approximate hydrophobicity was calculated according to the retention times of the chromatographic peaks. Next, the peptides were prepared at a concentration of 10 mg/mL and run on a semipreparative C-18 HPLC column. The purified peptide peaks were collected, and the samples were subsequently lyophilized (freeze-dried). The concentrations of the lyophilized AMP samples were determined using the Pierce Quantitative Fluorometric Peptide Assay kit (Thermo Fisher Scientific, Waltham, MA, USA).

### 2.2. Cytotoxicity Assay

The TIB-71 RAW 264.7 macrophage line, which was obtained from *Mus musculus*, was used in this study, and this cell line was supplied by ATCC (Manassas, VA, USA). The MTT Cell Proliferation Kit (Sigma–Aldrich, St. Louis, MO, USA), a colorimetric method, was used to evaluate the damage caused by the peptides in eukaryotic cells. TIB-71 RAW 264.7 macrophages were revived under appropriate culture conditions (37°C, 5% CO_2_) to prepare for the experiment. The final concentration of peptides ranged 0.125–64 μg/mL. Samples were examined in triplicates for each concentration. Cells incubated without peptide treatment under the same conditions were used as the control. Magainin 2 (Mag2), a known natural AMP with no cytotoxic effects, was used as a positive control [13]. The results were obtained using a 96-well plate reader at 550 and 690 nm.

### 2.3. Investigation of Tumor Necrosis Factor-*α* (TNF-*α*) Expression by ELISA

Within the scope of the ELISA study, the effect of varying concentrations of the peptides we synthesized on the mouse macrophage cell line was examined.

In this study, a cell line that was not treated with AMPs and LPS, a cell line treated only with AMPs, a cell line treated only with LPS, and a cell line treated with both LPS and AMPs were used. To find the changing TNF-*α* ratio after incubation, the kit named Mouse Tumor Necrosis Factor *α* ELISA Kit-RAB0477 (Sigma–Aldrich) was applied according to the protocol. Graphpad Prism (9.0.0) was utilized for performing a one-way ANOVA test (*α* = 0.05) for the statistical analysis.

### 2.4. Investigation of Interferon-*γ* (IFN-*γ)* Expression by ELISA

Similarly, ELISA was applied to measure the change in IFN-*γ* levels in response to the peptides and LPS. In this experiment, the Mouse IFN-*γ* ELISA Kit-E-EL-M0048 was applied according to the specified kit protocol, and the results were evaluated.

### 2.5. Interleukin-6 (IL-6) Expression Analysis by Western Blotting

Protein was extracted from TIB-71 cells for use in western blotting. For this purpose, the supernatant of the cells grown in the appropriate environment was separated for ELISA, whereas protein was extracted from the cellular pellets. Protein obtained using the Protein Extraction Kit (Abcam, Cambridge, UK) was prepared for western blotting. The concentrations of protein samples were determined using the Pierce BCA protein assay kit (Thermo Fisher Scientific). The results were analyzed by reading them on a 96-well plate reader at 565 nm.

IL-6 was separated by 12% (w/v) SDS-PAGE and transferred onto a PVDF membrane using the wet transfer technique to compare the expression levels of the markers. Proteins were measured using horseradish peroxidase (HRP)-based clarity western ECL substrate and read using ChemiDoc devices. The area of the bands obtained in the western blotting results was computed using Image Lab software.

Specifically, the volume occupied by the bands on the membrane was determined, and the band volume of IL-6 was normalized to that of *β*-actin to assess protein expression in a semiquantitative manner.

Microsoft Excel was used for the semiquantitative analysis. IL-6 secretion by mouse macrophages in response to AMP exposure was examined by GraphPad Prism 9.0.0 one-way ANOVA test (GraphPad, La Jolla, CA, USA).

## 3. Results

### 3.1. Sequences and Properties of the Designed AMPs

In this study, four peptide sequences, namely NET1, NET2, NET3, and NET4, were designed using d- and l-form amino acids. The properties of these peptides are presented shown in Table 1.

### 3.2. Chemical Synthesis of AMPs and Purification by HPLC

**Figure 1:**
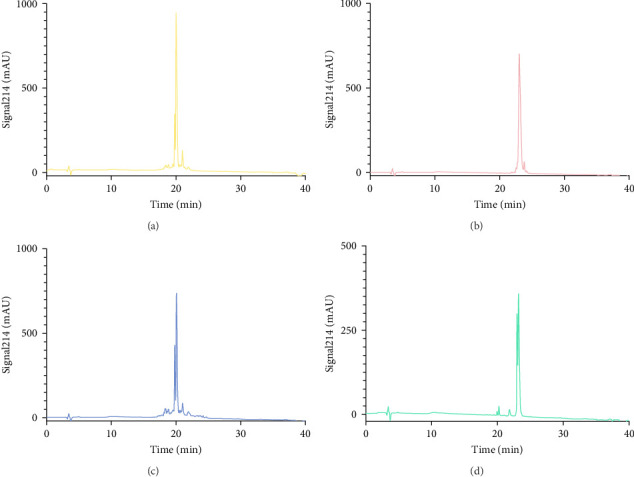
HPLC chromatograms. (A) NET1. (B) NET2. (C) NET3. (D) NET4.

The Liberty Blue peptide synthesizer was used for the chemical synthesis of peptides. In each synthesis, the amounts of amino acids used and the amount of dimethylformamide (DMF) dissolved in milliliters were calculated by the software of the peptide synthesizer. The peptide peaks appeared at 50%–55% hydrophobicity. Purity levels were determined by the ratio of peak areas to the total peak areas, and they were above 95%. In the analytical HPLC results, a single peak was observed for each peptide (Figure 1).

The concentrations of the peptides were measured using the Pierce Quantitative Peptide Assays kit. Once the peptide concentrations were determined, the peptides were stored under appropriate conditions for use in the experiments (Table 2).

### 3.3. Cytotoxicity Assay Results

The cytotoxicity of the synthesized peptides was determined in TIB-71 cells using the MTT method. The toxic effects of peptides at varying concentrations are extremely important for the vital activities of macrophages, which are members of the immune system.

**Figure 2:**
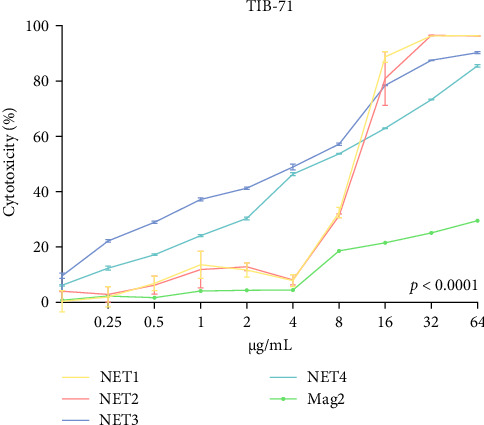
Percent cytotoxicity of NET1, NET2, NET3, NET4, and Mag2.

**Figure 3:**
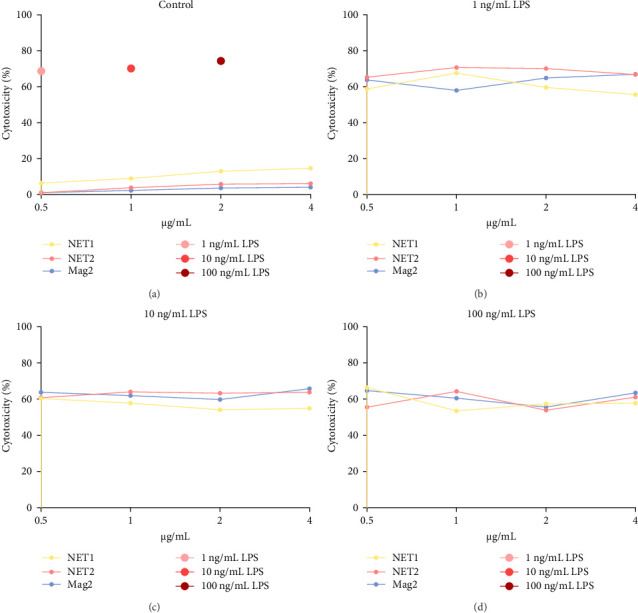
Percent cytotoxicity of NET1, NET2, and Mag2 in the presence of LPS. (A) Control cells with only NET1 peptides, respectively, and only LPS with 1, 10, 100 ng/mL concentrations. (B) Peptides and 1 ng/mL LPS. (C) Peptides and 10 ng/mL LPS. (D) Peptides and 100 ng/mL LPS. The *p*-value (probability value) for each graph is indicated in the figure.

The MTT assay was performed separately with and without LPS.  Figure 2 presents the results of cytotoxicity assays for various concentrations of the AMPs and Mag2. This experimental group was first tested in an LPS-free environment. NET1, NET2, and Mag2 did not cause significant cytotoxicity at concentrations up to 4 *μ* g/mL, and they were further evaluated in the presence of different LPS concentrations. The LPS-free wells were used as a control.  Figure 3 presents the percent cytotoxicity in the presence of different concentrations of LPS and the peptides.

### 3.4. Investigation of IFN-*γ* Responses

**Figure 4:**
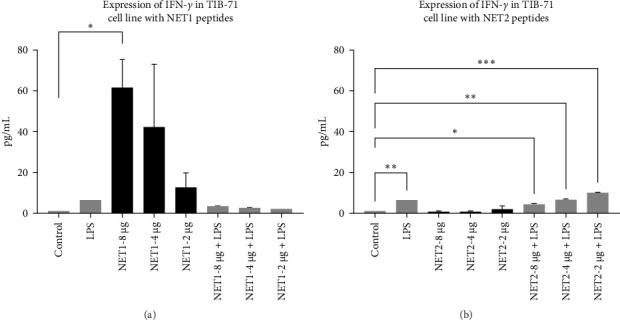
(A) IFN-*γ* secretion from TIB-71 macrophages in the presence of various concentrations of NET1 and LPS. (B) IFN-*γ* secretion from TIB-71 macrophages in the presence of various concentrations of NET2 and LPS.

ELISA was employed to assess IFN-*γ* secretion into the TIB-71 cell supernatant in the presence of LPS (50 ng/mL) in and increasing concentrations of the peptides. The significance of the change in expression was determined by one-way ANOVA. *p* < 0.05 indicated statistical significance. The graph showing the amount of IFN-*γ* in the samples is shown in Figure 4. The symbol “⁣^*∗*^” means *p* ≤ 0.05, the symbol “⁣^*∗∗*^” means *p* ≤ 0.01, and the symbol “⁣^*∗∗∗*^” means *p* ≤ 0.001.

### 3.5. Investigation of TNF-*α* Responses

**Figure 5:**
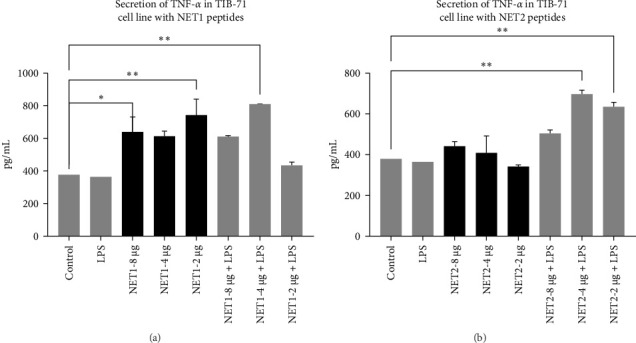
(A) Changes in TNF-*α* secretion from TIB-71 macrophages treated with NET1 and LPS. (B) Changes in in TNF-*α* secretion from TIB-71 macrophage cells treated with NET2 and LPS.

TNF-*α* secretion from TIB-71 macrophages in the presence of LPS (50 ng/mL) and various concentrations of the peptides was examined by ELISA. One-way ANOVA was used to determine whether the changes in TNF-*α* responses were statistically significant.  Figure 5 displays the concentrations of TNF-*α* in the samples.

### 3.6. Investigation of IL-6 Responses

**Figure 6:**
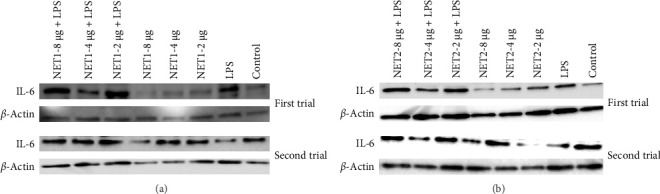
(A) IL-6 and *β*-actin bands at different concentrations of NET1 peptide and in the presence and absence of LPS. (B) IL-6 and *β*-actin bands at different concentrations of NET2 peptide and in the presence and absence of LPS. The study was repeated twice.

Images of the IL-6 bands obtained by western blotting and repeat probing of the membrane after stripping are presented in Figure 6.

**Figure 7:**
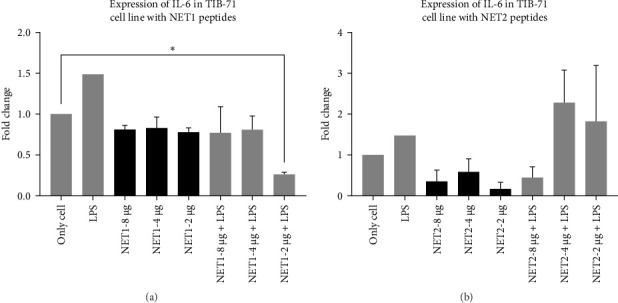
(A) Changes in IL-6 expression in TIB-71 macrophages in the presence of NET1 and LPS. (B) Changes in IL-6 expression in TIB-71 macrophages in the presence of NET2 and LPS.

IL-6 expression was normalized to *β*-actin expression and compared with the control (unstimulated cells). The relationship between different experimental groups was examined statistically by one-way ANOVA. In Figure 7, the fold changes in IL-6 expression in the presence of AMPs and LPS are presented.

## 4. Discussion

This research examined the antimicrobial activities of peptides based on helical structure, a crucial component of the immune system that is found in a wide variety of organisms. These peptides have important roles in natural defenses, and they simultaneously participate with the secondary immune response. The emergence of AMPs and their increasing importance in recent years are also related to the resistance of microorganisms to antibiotics, which has led to a need for alternative drug or molecule candidates. The AMPs used in this study include peptides previously developed and used by Polat et al. [10]. The peptides were resynthesized by solid-state synthesis and used in our experiments.

The 16-amino acid peptides used in this study were composed of two distinct amino acid types, namely the d- and l-forms. TIB-71 mouse macrophages were used in various tests to examine the effects of the peptides, the minimal inhibitory concentrations (MICs) of which in microorganisms were previously determined [10], on the immune system. We examined the interactions of the peptides with IL-6, IFN-*γ*, and TNF-*α*, which are important components structures of the immune system, through cytotoxicity assays, ELISA, and western blotting.

First, the peptides were synthesized and purified by HPLC, as presented in Figure 1. The appearance of a single peak indicated that the peptide was pure and confirmed the absence of external molecules. Fluctuations observed before purification indicate impurities.

These contaminants can be attributed to the fact that the purification and analysis arms of the device were not completely washed. In addition, the rate at which the peptide is introduced into the system during HPLC also causes contamination. To eliminate these impurities, we collected the peptides injected into the system after analyzing the impurity. Each peptide gave a single peak in the system, and it was collected back in pure form (Figure 1).

For cytotoxicity analysis, assays were conducted using macrophages, an immune system cell type that plays an effector role in many infectious diseases. In this context, TIB-71 mouse macrophages were chosen. The selectivity/safety index was calculated as a ratio of the MIC at which each AMP affects the microorganism to the concentration of AMP that causes 50% toxicity in the cells [10]. The important finding of this assessment was that AMPs exhibited both low inhibitory effects and low toxicity [14].

To conduct a broad screening, treatment with the peptides was started at a concentration of 64 µg/mL and reduced to 0.125 µg/mL. In this context, Mag2, which served used as a reference, exhibited low toxicity at almost all concentrations. NET1 and NET2 displayed similar cytotoxicity, starting at a concentration of 4 µg/mL. In particular, NET2 exhibited lower toxicity than the other peptides, even at high concentrations. Because of low concentrations of NET1 and NET2 compared with the other peptides, subsequent studies continued with these two peptides, and the concentration range was 0.5–4 µg/mL. LPS, which had a highly toxic effect alone, as expected, exhibited lower toxicity when applied in combination with the AMPs.

At a concentration of 4 µg/mL, NET1 displayed the lowest toxicity. The toxicity of NET1 was increased threefold compared to that in the absence of LPS. The level of toxicity of NET2 was increased by tenfold compared to that in the absence of LPS. There were no significant changes in the cytotoxicity depending on the LPS concentration. The cytotoxicity of NET2 and Mag2 increased as the LPS concentration decreased.

The results were similar when the peptide concentration was 2 µg/mL. The cytotoxicity of NET2 and Mag2 peptides increased with as the LPS concentration decreased. On average, NET1 displayed lower cytotoxicity than NET2 regardless of the LPS concentration. NET1 also exhibited lower cytotoxicity in samples in which the peptide concentration was 1 µg/mL. Similarly, in this analysis, the cytotoxicity of the peptides increased as the LPS concentration decreased.

Finally, in the analysis in which the peptide concentration was 0.5 µg/mL, decreased NET1 cytotoxicity was observed in response to decreasing LPS concentrations, whereas increased cytotoxicity was observed for NET2. Mag2 displayed a constant effect at all concentrations. This might be attributable to the slight mitogenic response to low levels of the peptide and LPS [15].

After cytotoxicity studies, ELISA was performed for NET1 and NET2 to assess IFN-*γ* secretion. In this context, the effects of the peptides both with and without LPS on IFN-*γ* secretion were investigated. The presence of LPS resulted in greater IFN-*γ* secretion than observed in unstimulated cells. When cells were treated with NET1 at three different concentrations in LPS-free medium, IFN-*γ* secretion increased significantly. IFN-*γ* secretion decreased as the peptide concentration decreased. When cells were treated with NET1 in the presence of LPS, a decrease IFN-*γ* expression occurred. Consequently, the secretion of IFN-*γ* also decreased. The expression of IFN-*γ* in cells stimulated with 8 µg/mL NET1 increased significantly at three different concentrations compared with the control level.

Meanwhile, NET2 induced lower IFN-*γ* expression than NET1. Moreover, with increasing concentrations of NET1, IFN-*γ* secretion increased, whereas the opposite was observed for NET2. Furthermore, in contrast to NET1, the IFN-*γ* response was stronger when NET2 and LPS were used as costimulators. In effector cells stimulated with NET2 and LPS, IFN-*γ* secretion was significantly increased.

Normally, the major sources of IFN-*γ* expression are T lymphocytes and NK cells. However, some studies revealed that this situation can change, and IFN-*γ* can be secreted by macrophages or different structures [16]. This study illustrated that NET1 and NET2 significantly increased IFN-*γ* secretion, and NET2 displayed strong stimulatory properties in the presence of LPS. This situation, in which the effect changed in the presence and absence of LPS, was conspicuous in terms of its pro-inflammatory effect. Cytokine secretion, which becomes evident during acute inflammation, is effective against many infections. Studies should also be conducted with different cytokines to observe the immune effect of these peptides against various diseases. IFN-*γ* is involved in immune response modulation, including host defense against intracellular infections and tumors. IFN-*γ* facilitates Th1 differentiation, enhances macrophage activity, promotes leukocyte migration to the infection site, and increases major histocompatibility complex expression to improve T-cell identification of cancerous or infected cells. Nonetheless, uncontrolled IFN-*γ* expression in mice induces autoimmunity, and unchecked IFN-*γ* protein synthesis is likewise harmful to the host. Consequently, maintaining a balance between immunological tolerance and inflammation requires strict control over IFN-*γ* expression [17–19].

Cytokine secretion, which comes to the fore during acute inflammation, is effective against many infections. Studies should also be conducted with different cytokines to observe the immune effect of these peptides against diseases. Although their main job is to control inflammation, cytokines are essential for controlling the immune system in both health and illness. Cytokines are classified as either pro- or anti-inflammatory [20].

In this study, we examined the effects of NET1 and NET2 on TNF-*α* expression at the cellular level in the presence and absence of LPS. There was no change in its expression in cells exposed only to LPS compared with the control. Different concentrations of NET1 in LPS-free medium significantly increased TNF-*α* secretion by 50%. However, there was no significant difference according to the peptide concentration. In addition, the TNF-*α* response did not differ between cells simultaneously stimulated with the peptide and LPS and cells treated only with the peptide.

Similar results emerged in studies conducted with NET2. TNF-*α* secretion decreased with decreasing peptide concentrations in LPS-free medium. In the presence of LPS, the TNF-*α* response is stronger than that observed in the NET1 group. TNF-*α* secretion increased significantly, especially in the presence of 2 or 4 µg/mL NET2 combined with LPS. Studies conducted using lower peptide concentrations in an LPS-free environment have the potential to reveal anti-inflammatory effects [21–23].

The findings illustrated that NET1 and NET2 might have anti-inflammatory effects. The results were evaluated on the basis of the amount of IL-6 released from the cells into the medium alone. In this context, the amount of IL-6 released from LPS-treated cells increased by 50%, consistent with the data in the literature [21, 24]. The long-term presence of IL-6 can cause chronic inflammation. Because of cellular treatment with NET1, the amount of IL-6 released into the medium was decreased. It is predicted that the peptides have anti-inflammatory effects. However, no concentration-dependent change was observed. The same result was obtained when the cells were treated with LPS alone, namely, decreased IL-6 expression. An important point is that at the lowest concentration of 2 µg/mL, IL-6 expression was threefold lower than observed in the other groups.

Similar results were obtained for NET2. When NET2 is used alone, IL-6 expression was 50% lower than that observed in NET1-treated cells. However, there was no significant change in IL-6 as the peptide concentration was decreased. When cells treated with LPS were then treated with NET2, an anti-inflammatory effect was recorded at a peptide concentration of 8 µg/mL. However, as the concentration was decreased, this effect disappeared, and IL-6 expression increased significantly. Both peptides induced the highest IL-6 expression at a concentration of 4 µg/mL in both the presence and absence of LPS. IL-6 expression was significantly increased by both 2 and 4 µg/mL NET2 in combination with LPS in comparison to the findings in the other groups. This might have been caused by the aggregation of peptides at low concentrations or disruption of peptide structures upon stimulation with LPS. Detailed studies should be conducted using a wide range of concentrations.

The aims of the study were to compare the effects of our synthesized peptide structures on the immune system and evaluated their effectiveness. Many AMPs are promising structures for the treatment of various diseases with two-way effects on the immune system, as they exhibit both pro-inflammatory and anti-inflammatory effects, especially in response to inflammation. In this context, NET1 and NET2 exhibited anti-inflammatory effects by decreasing IL-6 and TNF-*α* at certain concentrations, whereas pro-inflammatory effects were induced through IFN-*γ*. This situation is compatible with the literature, and by increasing the diversity of experiments and improving the peptide structures, new therapeutic structures could be generated.

In addition, the use of some AMPs alone or in combination gave better results on immune system elements. For example, it has been observed that LL-37 peptide, used in combination as an innovative approach in ovarian cancer, has an antitumor effect and increases the activity of NK cells [25]. In another example, macrophage transformation and effects of monocytes were examined using various stimuli in the presence of LL-37. Monocytes cultured with M-CSF were directed to macrophages with pro-inflammatory properties [26]. A further instance of macrophage turnover and the balance of pro-inflammatory and anti-inflammatory cytokines, LL-37 peptide induced anti-inflammatory IL-10 while reducing TNF-*α* and IL-17. It also affected the production of TGF-*β* [27]. Inflammatory effects are also observed in peptides that are not natural but produced in mutant form. For example, mutated chensinin-1 peptide blocked LPS-induced pro-inflammatory cytokines [28]. In another example of structurally changed peptides, pseudin-2 peptide isolated from *Pseudis paradoxa* was structurally changed and its anti-inflammatory effects were examined [29]. Also full Ala-scan can also be performed to reveal the effect on cells. Performing these tests can provide detailed insights into the effects of AMPs against pathogenic cells. The interactions between AMPs and cells are crucial for modifying the pathways needed to neutralize pathogens, including chemical, biological, and electrostatic interactions. Changes and improvements in the primary and secondary structures of peptides directly influence their effectiveness against pathogenic cells. Additionally, investigating the D-Ala-D-Ala structures, which are vital components of bacterial peptidoglycan and play a role in antibiotic resistance mechanisms, is essential. Modifying synthetically produced AMPs based on their mechanisms of action is also critical for developing innovative treatment products against pathogenic microorganisms. Furthermore, conducting a full Ala-scan could further elucidate the impact of these peptides on cells.

In this context, the NET1 and NET2 peptides we synthesized cause anti-inflammation at certain concentrations against IL-6 and TNF-*α*, while IFN-*γ* also causes pro-inflammation. This situation is compatible with the literature, and by increasing the diversity of experiments and improving the peptide structures we have obtained, it can lead to new generation therapeutic structures.

## 5. Conclusion

Based on the experimental results, we believe it is important to apply these peptides over a wider concentration range and determine their effects on different immune system elements. Investigating the effects on the AMPs on different IL molecules working together simultaneously or evaluating their effects on different cytokine groups could expand their applicability. Animal studies should also be conducted to investigate the effects and toxicity of the peptides at different doses.

The combined application of synthesized peptides could reveal product groups with high commercialization potential through studies conducted under both in vitro and in vivo conditions.

## Figures and Tables

**Table 1:** Designed peptide sequences and their properties [10].

	Peptide sequences	Amino acid content
NET1	RLLLRLLRRLLRLLLR-NH_2_	D-leucine, L-arginine
NET2	RLLLRLLRRLLRLLLR-NH_2_	L-leucine, L-arginine
NET3	RLLLRLLRRLLRLLLR-NH_2_	L-leucine, D-arginine
NET4	RLLLRLLRRLLRLLLR-NH_2_	D-leucine, D-arginine

**Table 2:** Stock peptide concentrations after HPLC.

Peptide	Peptide concentration (mg/mL)	Absorbance	Peptide concentration (µm)
NET1	1.65	19.72	795.16
NET2	1.5	17.85	719.75
NET3	1.87	22.34	900.80
NET4	1.71	20.43	823.79
Mag2	2.14	21.56	869.35

Abbreviations: HPLC, high-performance liquid chromatography; Mag2, magainin 2.

## Data Availability

Dataset is available on request from the authors.
